# Transepithelial corneal cross-linking: a review

**DOI:** 10.1007/s10792-025-03928-1

**Published:** 2026-01-10

**Authors:** Wen Zhou, Sandeepani K. Subasinghe, Francesc March de Ribot, Kelechi C. Ogbuehi, George J. Dias

**Affiliations:** 1 Department of Anatomy, University of Otago, 270 Great King Street, P.O. Box 913, Dunedin, 9054 New Zealand; 2 Department of Medicine, Dunedin School of Medicine, University of Otago, Dunedin, New Zealand; 3 Faculty of Medicine and Health, University of Sydney, Sydney, NSW Australia

**Keywords:** Keratoconus, Crosslinking, Transepithelial corneal cross-linking, Riboflavin penetration, Corneal epithelium

## Abstract

**Purpose:**

This review aims to summarize the current understanding of transepithelial corneal cross-linking (TE-CXL) for treating keratoconus (KC). It focuses on how TE-CXL compares with the standard epithelium-off cross-linking (S-CXL) and discusses recent improvements intended to make it more effective.

**Methods:**

Relevant studies were reviewed from PubMed and Google Scholar. The review focused on research about new riboflavin solutions, delivery techniques, ultraviolet-A (UV-A) light settings, oxygen supply methods, and recent new technologies designed to improve the results of TE-CXL.

**Results:**

TE-CXL preserves the corneal epithelium, providing better patient comfort and fewer postoperative complications. However, its corneal stiffening effect is generally lower than S-CXL due to limited riboflavin penetration and UV photoactivation. Recent approaches, including chemical enhancers, iontophoresis-assisted delivery, optimized UV-A protocols, nanotechnology-based or ultrasound-assisted methods have demonstrated potential to improve biomechanical strengthening. In addition, theranostic-guided TE-CXL, which provides real-time monitoring of stromal riboflavin concentration and adaptive UV-A dosing, represents a promising advancement. Nevertheless, differences in treatment protocols and in oxygen and luminance parameters still lead to variability in clinical outcomes.

**Conclusions:**

TE-CXL is a promising and less invasive treatment for KC, offering better comfort and faster recovery. However, its long-term stability and biomechanical effect remain inferior to S-CXL. Future progress will depend on optimizing riboflavin and oxygen delivery, refining UV-A irradiation protocols, and validating newer technologies such as theranostic-guided CXL through large-scale clinical studies.

## Introduction

Keratoconus (KC) is a progressive corneal ectatic disorder characterized by bilateral thinning of the central, paracentral, or peripheral corneal tissue [1]. Although KC was traditionally considered a non-inflammatory condition, increasing evidence indicates that inflammatory and oxidative-stress–related pathways may contribute to its onset and progression [2]. This thinning leads to irregular astigmatism, progressive myopia, and an increase in higher-order aberrations (HOAs), which can ultimately result in significant vision loss [3, 4]. KC usually develops around puberty, but it is most commonly diagnosed in the second and third decades of life. The disease progressively worsens into the fourth decade, after which the progression usually stops, and the condition stabilizes [5]. Epidemiological studies estimate the prevalence of KC to range from 0.2 to 4,790 per 100,000 individuals, particularly in Asian populations [6, 7]. Recent studies using advanced imaging suggest KC prevalence is higher than previously reported, with significant variability across populations [8, 9]. Population-based studies using Scheimpflug imaging and Placido-disk topography report higher prevalence rates and can even detect earlier stages of keratoconus, indicating that earlier estimates substantially underestimated the true disease burden [10, 11].

Corneal cross-linking (CXL) is a treatment developed to address the progression of the KC by re-strengthening the corneal collagen matrix, ultimately reducing or delaying the need for corneal transplantation. This treatment uses ultraviolet-A light and riboflavin, inducing the production of reactive oxygen species, which facilitate the formation of additional covalent bonds between collagen molecules [12]. This CXL process enhances the rigidity and stability of the corneal structure. The standard epithelium-off cross-linking (S-CXL) approach, commonly referred to as the Dresden protocol, removes the central corneal epithelium to facilitate adequate diffusion of riboflavin and oxygen into the stroma [13–16]. Although S-CXL remains the clinical standard due to its well-established efficacy in halting KC progression, epithelial removal is associated with postoperative pain, delayed epithelial healing, and a higher risk of infection and stromal haze, both of which are relatively uncommon complications. [17–25]. Therefore, although S-CXL is the most widely performed and accepted standard worldwide, the development of transepithelial CXL (TE-CXL) has generated ongoing debate regarding its efficacy and long-term outcomes. With the introduction of TE-CXL, the epithelium remains intact during the CXL procedure, offering advantages such as faster healing, reduced discomfort, quicker visual recovery, and a lower risk of corneal infection [26]. Despite these potential benefits, TE-CXL presents with several technical challenges, including limited riboflavin penetration, reduced UV penetration, and restricted oxygen diffusion due to the intact epithelial barrier, raising concerns about achieving adequate CXL treatment depth [27, 28]. Several meta-analyses have explored the efficacy of TE-CXL compared to S-CXL. Most systematic reviews indicate that TE-CXL is generally less effective than S-CXL in arresting KC progression and providing biomechanical reinforcement [29–31]. A small numbers of studies have reported comparable outcomes between S-CXL and TE-CXL, particularly with improvements in corrected distance visual acuity (CDVA) [32–34]. However, the preponderance of evidence favors S-CXL, which remains the reference standard pending further high-quality comparative trials. These conflicting findings highlight the need for further high-quality randomized controlled trials to clarify the comparative efficacy of TE-CXL and S-CXL. Despite this, TE-CXL techniques are particularly appealing because they avoid epithelial removal, reducing associated risks and enabling faster postoperative recovery.

This review provides a comprehensive summary of TE-CXL, focusing on its current applications, clinical outcomes, commonly used techniques, and recent advancements. By reviewing existing evidence, this article aims to clarify the role of TE-CXL in managing KC and evaluate its potential as a safer but at least equally effective alternative to S-CXL.

## TE-CXL

TE-CXL technique is a modification of the S-CXL procedure that aims to preserve the corneal epithelium during treatment. This approach offers several significant advantages by eliminating the need for epithelial removal. It reduces postoperative discomfort and minimizes associated risks such as infection and delayed healing. Additionally, TE-CXL facilitates a faster return to baseline vision and the resumption of contact lens use, in return enhancing patient convenience and quality of life. Patients also experience a shorter duration of discomfort. The procedure carries a lower risk of corneal infections and haze compared to S-CXL, making it a safer and more patient-friendly alternative [35]. The concept of TE-CXL was first introduced in 2004 by Boxer Wachler in the United States, who explored the possibility of performing riboflavin-UVA CXL without epithelial removal [36]. The idea was later adopted in Europe by Pinelli in 2006, who further refined the technique and contributed to its clinical application [37]. Although the epithelium does not block the passage of UVA, it prevents sufficient riboflavin from penetrating into the corneal stroma because of its tight junctions and lipophilic barrier [38]. Consequently, an intact epithelium often compromises the effectiveness of CXL, making riboflavin delivery into the corneal stroma a significant challenge in TE-CXL [39].

### Role of the epithelium

The corneal epithelium is the outermost layer of the cornea and constitutes approximately 10% of the total corneal thickness. It serves as a critical barrier and regulatory interface. It is composed of 4 to 6 layers of non-keratinized, stratified squamous epithelial cells (basel, wing, and superficial cells) with a thickness of 40–50 μm [40–42]. Neighboring cells are connected by desmosomes and anchored to the basement membrane through hemidesmosomes and anchoring filaments [43]. This tight intercellular junctions create a lipophilic barrier that blocks most hydrophilic molecules, including riboflavin, while maintaining stromal hydration and protecting against microbial and chemical insults [44, 45]. The corneal epithelium is a regenerative tissue that renews itself completely within a week, driven by the mitotic activity of basal cells [46]. This rapid healing capacity allows for the safe removal of the epithelium in S-CXL, ensuring effective riboflavin penetration and sufficient CXL depth.

**Fig. 1:**
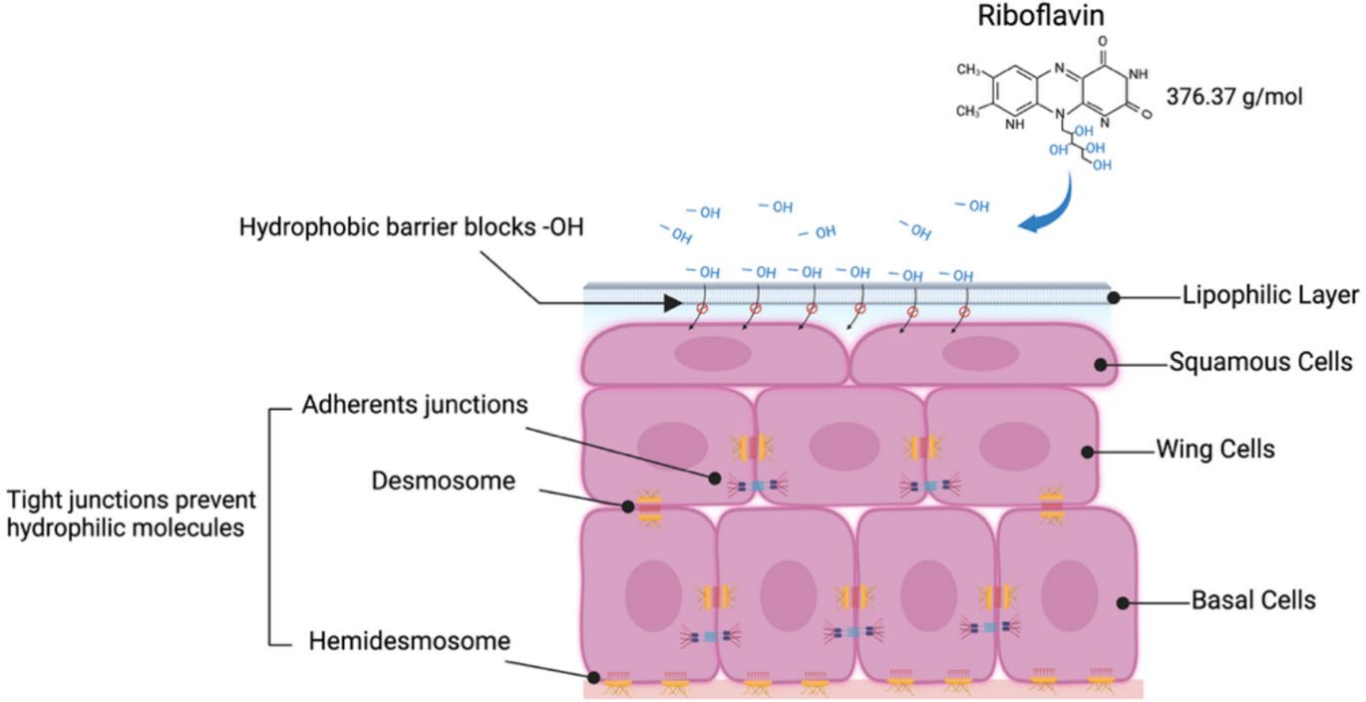
Corneal epithelium as a hydrophobic barrier preventing riboflavin penetration

**Fig. 2:**
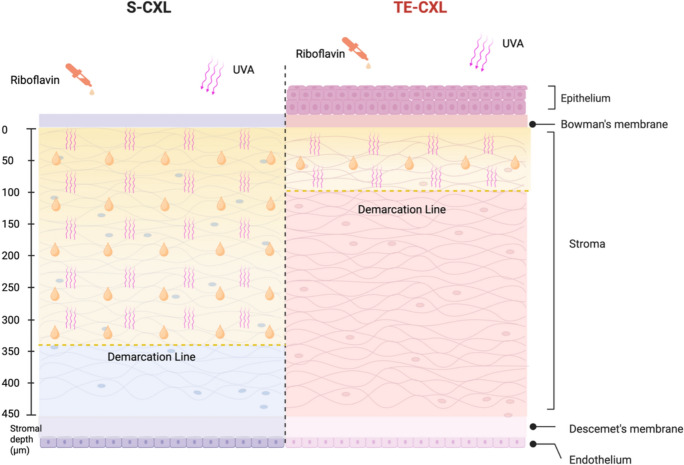
Comparison of S-CXL and TE-CXL in terms of riboflavin penetration, UVA exposure, and CXL depth

One of the defining features of TE-CXL is the preservation of the corneal epithelium, which eliminates the risks associated with epithelial removal. However, the intact epithelium also presents significant challenges, particularly in terms of riboflavin penetration. As a hydrophilic molecule, riboflavin diffuses poorly through the lipophilic epithelial barrier, and tight junctions further restrict its passage [47]. Additionally, the tight junctions between epithelial cells further restrict the diffusion of hydrophilic molecules (Fig. 1). As a result, the lipophilic corneal epithelium markedly reduces riboflavin permeability and also limits oxygen availability, which is essential for the CXL reaction [48]. Previous studies have shown that stromal riboflavin levels increase only when the epithelium is removed, and the resulting demarcation line in TE-CXL is consequently much shallower than in S-CXL **(**Fig. 2) [27, 49].

During TE-CXL, the corneal epithelium provides some protection against UV-A due to its antioxidant content [50]. Although it absorbs UV-B (wavelengths 280–330 nm) more strongly than UV-A (365 nm), the UV-A used in CXL is largely transmitted to the stroma [51, 52]. Thus, the epithelium only partially attenuates UV-A, and its main impact on TE-CXL efficacy is through restricting riboflavin and oxygen diffusion rather than blocking UV irradiation. Ensuring adequate stromal riboflavin loading remains essential for achieving effective CXL [38].

### Impact on endothelium

The corneal endothelium is a single layer of endothelial cells lining the inner surface of the cornea. Its primary functions are to maintain corneal transparency and control nutrient exchange [53]. The corneal endothelial cell density (ECD) in adults is approximately 3000 cells/mm^2^, gradually decreasing at an average rate of about 0.6% per year until it stabilizes around the age of 50 [54, 55]. Because human corneal endothelial cells are essentially non-regenerative, cell loss is compensated mainly by cellular enlargement [56]. Once the ECD falls below a critical range of approximately 400–500 cells/mm^2^, endothelial decompensation may occur, leading to progressive corneal edema and visual loss, for which corneal transplantation is often required [57, 58].

Endothelial damage is a serious potential complication of CXL, particularly in thin corneas. During standard S-CXL, stromal riboflavin absorbs most of the UV-A and reduces the dose reaching the endothelium. However, when the cornea is too thin, insufficient attenuation may expose the endothelium to cytotoxic irradiance levels [59, 60]. Therefore, a minimum S-CXL corneal thickness of 400 μm has traditionally been recommended as a safety threshold. However, endothelial damage has still been reported in cases where the cornea exceeded 400 μm [61–63]. Additionally, the use of standard dextran-riboflavin solutions has been shown to cause approximately 9.08% corneal thinning [64, 65], and deeper riboflavin penetration with frequent instillation may further increase the risk by enabling ROS generation closer to the endothelium [66]. Recent evidence suggests that the traditional 400 μm safety threshold may be overly conservative, as some studies have shown that controlled CXL protocols can be performed safely in slightly thinner corneas [67]. However, excessively thin corneas still pose a real risk of endothelial overexposure and potential cell damage. In contrast, TE-CXL offers an additional protective mechanism because the intact epithelium absorbs and temporarily retains riboflavin, helping attenuate UV-A transmission and reducing the risk of endothelial exposure [68]. In summary, deeper riboflavin penetration improves the biomechanical effect of CXL but increases risk to deeper structures, whereas shallower penetration is safer but may compromise treatment efficacy.

## Different epi-on CXL approaches

One of the primary challenges in TE-CXL is ensuring sufficient riboflavin penetration through the intact corneal epithelium. To address this, a wide range of strategies have been developed, aiming to enhance the efficacy of TE-CXL.

### Chemical strategies and advanced riboflavin formulations for enhanced penetration

#### Benzalkonium chloride (BAC)

The results of clinical studies on using chemical enhancers for TE-CXL have been mixed, making it hard to compare them because there are no consistent standards (Table 1). The primary approach to enhancing riboflavin penetration currently involves the use of chemical enhancers, with BAC being one of the most commonly employed agents [69]. It is widely used as a preservative in eye drops at concentrations ranging from 0.0075 to 0.02%, and it can loosen the tight junctions of the corneal epithelium, enhancing drug permeability. Initially, Wollensak et al. conducted BAC-assisted TE-CXL experiments on rabbits [70]. They pretreated the cornea with 0.5% proparacaine eye drops and 0.005% BAC, observing a significant increase in corneal stiffness, though the effect was only one-fifth as effective as S-CXL. This result was later validated in clinical trials, where Koppen et al. attempted to use 0.005% BAC, but the results showed only an improvement in CDVA and a significant progression in maximum keratometry (Kmax) [71]. Building on the protocol suggested by Pinelli, which used 0.02% BAC, Kissner et al. evaluated the efficacy of 0.04% BAC and 0.02% BAC for TE-CXL in rabbits [72, 73]. Their findings confirmed that increasing the BAC concentration did not lead to greater corneal stiffening. Further studies by Raiskup et al. compared the effects of 0.44% and 0.90% NaCl solutions containing 0.02% and 0.01% BAC on riboflavin permeability. The results suggested that riboflavin solutions should contain 0.01% BAC and 0.44% NaCl to optimize riboflavin penetration [74]. Therefore, the concentrations of BAC used in clinical applications are primarily around 0.01%. It is typically administered in the form of proparacaine eye drops containing 0.01% BAC every five minutes for a total duration of 30 min prior to the CXL procedure to enhance the permeability of the epithelium to riboflavin [75–79]. This improved penetration has been shown in clinical studies to stabilize the Kmax value or reduce corneal steepening, enhancing the overall effectiveness of the procedure.

**Table 1:** Clinical outcomes of chemically enhanced TE-CXL

Authors	Year	Participants/Eyes	Follow-up	Type of study	Type of control group	Treatment for TE-CXL	Key results	ECD
Roszkowska AM, et al. [111]	2024	50/50	12 months	Prospective	S-CXL	RitSight + theranostic-guided TE-CXL	60% of eyes achieving > 1.0 D of flattening	Unchanged
Lombardo M, et al. [112]	2025	3/6	24 months	Case seriess	Treated eye	RitSight + theranostic-guided TE-CXL	All patients showed Kmax flattening ≥ 3.8 D	No reported
Koppen C, et al. [71]	2012	38/53	18 months	Prospective	Treated eye	Ricrolin + 0.5% proparacaine + 0.005% BAC	Only CDVA improved	No reported
Peter S Hersh, et al. [75]	2018	56/82	12 months	Prospective	Treated eye	0.1% riboflavin + proparacaine containing BAC 0.01%	Kmax decreased by 0.45D ± 1.94D (*P* = 0.04); UDVA improved (*P* = 0.02)	No reported
J Lai M, et al. [76]	2020	43/59	12 months	Prospective	Treated eye	0.1% riboflavin + proparacaine containing BAC 0.01%	Corneal haze increased initially, then decreased, with no impact on outcomes	No reported
Rozema JJ, et al. [77]	2013	25/35	6 months	Prospective	S-CXL and healthy myopic eyes	0.1% riboflavin + 20% dextran + 0.5% proparacaine + 0.01% BAC	S-CXL increases stromal backscatter; forward scatter increases similarly in both	No reported
Beckman KA [78]	2021	17/25	12 months	Prospective	Treated eye	0.146% riboflavin + proparacaine with BAC 0.01%	Kmax was stable. UCVA and CDVA improved	No reported
Lesniak SP, et al. [79]	2014	25/30	6 months	Prospective	Treated eye	0.1% riboflavin + proparacaine containing BAC 0.01%	Kmax and CDVA improved	No reported
Franch A, et al. [82]	2015	12/12	After treated	Prospective	S-CXL and I-CXL	Ricrolin +	Intrastromal riboflavin concentration in TE was 7 times lower than in S-CXL	No reported
Heikal MA, et al. [94]	2017	18/30	12 months	Prospective	Treated eye	ParaCel + 0.22% riboflavin	CDVA, SE, Ast and K values improved	No reported
Rush SW, et al. [95]	2017	131/131	24 months	Prospective	S-CXL	MedioCross TE	Kmax has greater improvement in S-CXL verse TE-CXL	No reported
Mesen A, et al. [97]	2018	124/124	24 months	Prospective	S-CXL and A-CXL	MedioCross TE	S-CXL and A-CXL have deeper DD than TE-CXL	No reported
Akbar B, et al. [100]	2017	64/64	18 months	Prospective	S-CXL	Peschke TE	Kmax, Steep K, and Sim K reduced in S-CXL. TE-CXL has 25% continued progression	No reported
Leccisotti A, et al. [113]	2010	51/51	12 months	Prospective	Untreated eye	Ricrolin	CDVA improved, SER decreased, Kapex and ISV increased	unchanged
Buzzonetti L, et al. [114]	2012	13/13	18 months	Prospective	Treated eye	0.1% riboflavin + Tris + EDTA	CDVA improved, but K readings and HOAs worsened significantly in children	No changed
Malhotra C, et al. [115]	2022	49/58	24 months	Retrospective	S-CXL and CACXL	Peschke TE	CACXL and TECXL were comparable with the S-CXL	No reported
Akram S, et al. [116]	2022	18/32	5 years	Retrospective	Treated eye	1% riboflavin	BCVA and Kmax improved	No reported
Eraslan M, et al. [117]	2017	27/36	24 months	Prospective	S-CXL	MedioCross TE	TE-CXL is about 70% as effective as S-CXL in stopping KC progression	Unchanged
EI-Kateb M, et al. [118]	2017	26/48	12 months	Prospective	S-CXL and A-CXL	MedioCross TE	Cornea thinned. UDVA, CDVA, K value remained stable. S-CXL was better than TE-CXL	No reported
Chen SH, et al. [119]	2016	16/16	12 months	Prospective	Untreated eye	0.5% riboflavin + 0.1% Tetracaine	UDVA, CDVA, SE, Kmax improved significantly	Decreased insignificantly
Nawaz S, et al. [120]	2015	40/40	6 months	Prospective	S-CXL	Nano XL + 0.5% proparacaine	CDVA, Kmax, CCT improved significantly	Unchanged
Al Fayez MF, et al. [121]	2015	70/70	3 years	Prospective	S-CXL	0.1% riboflavin + 1% tetracaine + 0.02% BAC	Kmax increased by 1.1 D; 55% showed KC progression in TE-CXL	Unchanged
Hassan ME [122]	2013	15/15	6 months	Prospective	S-CXL	0.1% riboflavin + 20% dextran	The TE-CXL technique is easier, more comfortable for patients, and reduces postoperative corneal haze	No reported
Stojanovic A, et al. [123]	2012	53/61	12 months	Retrospective	Treated eye	0.5% riboflavin + BAC-containing local medication	UDVA, CDVA, Kmax, and SE improved	Decreased insignificantly

Min = minutes; TE-CXL = transepithelial corneal crosslinking; S-CXL = standard epithelium-off cross-linking; CACXL = contact lens-assisted corneal crosslinking; I-CXL = transepithelial epi-on by iontophoresis; A-CXL = accelerated corneal crosslinking; RitSight: 0.22% riboflavin; Ricrolin: 0.1% riboflavin + 20% dextran T500; Ricrolin + : 0.1% riboflavin + 0.1% EDTA + 0.05% Tris + Bihydrate Sodium Phosphate Monobasic & Bibasic; MedioCross TE: 0.25% riboflavin in 1.2% methylcellulose (HPMC) with 0.01% BAC; Nano XL: 0.1% riboflavin + 20% dextran; ParaCel: 0.25% riboflavin in HPMC with 0.02% BAC, EDTA; Peschke TE: 0.25 riboflavin in 1.2% HPMC with 0.01% BAC; D = diopter; Kmax = maximum keratometry; UDVA = uncorrected distance visual acuities; CDVA = corrected distance visual acuities; Kmean = mean K, SER = spherical equivalent refraction, Kapex = curvature at apex on tangential map; ISV = index of surface variance; HOAs = higher-order aberrations; Steep K = keratometry at steep axis; ECD = endothelial cell density; Ast = astigmatism; DD = demarcation line depth; CCT = central corneal thickness

#### Ethylenediaminetetraacetic acid (EDTA)

EDTA, a commonly used chelating agent, was originally developed for dialysis but is now widely included in ophthalmic formulations as a preservative and permeability enhancer [80]. In CXL, EDTA chelates calcium ions, reducing epithelial cohesion and enhancing the permeability of riboflavin. A study evaluating the effects of BAC and EDTA, either alone or in combination, on the diffusion of the hydrophilic molecule acyclovir (ACV) across the cornea in rabbits found that the combined use of BAC and EDTA significantly enhanced ACV permeability [81]. This finding provides a theoretical basis for combining both agents to facilitate the effective penetration of hydrophilic molecules like riboflavin in TE-CXL. Currently, there is no standardized concentration for EDTA in clinical use, and its dosage is typically determined by the surgical protocol. For example, Franch et al. utilized Ricrolin + in TE-CXL, which contained 0.1% riboflavin, 0.1% EDTA, 0.05% Tris, and sodium phosphate monobasic and bibasic. However, their results showed that the intrastromal riboflavin concentration in TE-CXL was seven times lower than that achieved in S-CXL procedures, indicating limited penetration [82]. An animal study by Armstrong BK et al. documented that BAC-EDTA TE-CXL caused less stromal cell death and reduced the risk of endothelial cell damage compared to S-CXL [83]. Additionally, the biomechanical stiffening effect produced by BAC-EDTA TE-CXL was found to be greater than that of S-CXL [69]. However, the toxicity of BAC and EDTA cannot be overlooked. Even at a low concentration of 0.001%, BAC exposure for more than 30 min can damage corneal epithelial cells, causing cytoplasmic injury [84]. This highlights the need for careful control of concentration and length of exposure to minimize toxicity while maintaining the efficacy of chelating agents [85, 86].

#### Hydroxypropyl methylcellulose (HPMC)

HPMC has been shown to maintain corneal thickness while preserving stromal riboflavin distribution [87]. Both HPMC and dextran function as viscosity agents to maintain osmolarity and enhance UVA absorption. However, clinical studies have shown that HPMC-based solutions provide better demarcation line clarity and improved biomechanical outcomes compared with dextran-based formulations [88]. Although one animal study reported slightly stronger biomechanical responses with dextran-based formulations, this was likely related to differences in experimental conditions [89]. Riboflavin solutions containing dextran have also been shown to significantly reduce corneal thickness during CXL, whereas HPMC-based solutions have little impact on corneal thickness [90, 91]. However, although HPMC helps maintain corneal hydration, studies show that HPMC-based formulations still allow only limited riboflavin penetration because of their high viscosity. Recent evidence indicates that increased stromal hydration under HPMC does not necessarily lead to a shallower cross-linked depth. In fact, swelling-induced oxygen availability may result in deeper or even excessive CXL, suggesting a potential risk of over-CXL under certain conditions [92]. HPMC also enhances the diffusion of hydrophilic molecules and is frequently combined with penetration enhancers such as BAC, Tris, or EDTA to facilitate riboflavin penetration [93]. As a result, HPMC-based riboflavin formulations have become widely used in current clinical practice where maintaining corneal thickness is essential.

#### Advanced riboflavin formulations

A variety of modified riboflavin formulations have been developed incorporating HPMC and these enhancers to optimize the TE-CXL process. Examples include several commercial products such as MedioCross TE, containing 0.25% riboflavin in 1.2% HPMC with 0.01% BAC. Other formulations include ParaCel, which combines 0.25% riboflavin with HPMC, 0.02% BAC, and EDTA, and Peschke TE, which also contains 0.25% riboflavin in 1.2% HPMC with 0.01% BAC. These formulations have been investigated in clinical studies, demonstrating their potential to achieve effective riboflavin saturation in the stroma while maintaining corneal safety and improving outcomes in TE-CXL [94–97]. An ex vivo study assessing riboflavin absorption found that MedioCross TE achieved higher stromal riboflavin concentrations compared to other formulations. However, its penetration remained lower than that observed in S-CXL [98]. Postoperative epithelial integrity remains a concern, with epithelial defects observed on day one in 50% of eyes treated with ParaCel alone and in most cases using MedioCross TE [99]. Moreover, a study involving Peschke TE reported that 25% of cases exhibited continued disease progression at the one-year follow-up, suggesting that TE-CXL may still face challenges in achieving sufficient biomechanical stability in certain cases [100]. Despite advancements in formulation design, some studies have demonstrated that simply removing dextran from riboflavin solutions or adding chemical enhancers, such as BAC, EDTA, Tris or NaCl, does not significantly enhance riboflavin penetration through an intact corneal epithelium [101, 102]. Although NaCl may influence osmolarity and epithelial hydration, its effect on permeability enhancement appears limited and inconsistent. Further refinements in formulation and technique may be necessary to address these limitations and improve long-term outcomes.

In addition to these, two novel riboflavin formulations designed specifically for TE-CXL, Ribocross TE and Ribostat CXLO, are under clinical investigation with promising results. Ribocross TE utilizes D-Alpha-tocopheryl poly (ethylene glycol) 1000 succinate (VE-TPGS) as a permeability enhancer, which is widely recognized as a non-ionic surfactant that facilitates drug absorption across various biological barriers. In addition to enhancing ocular permeability, VE-TPGS also protects cell membranes from free radical damage [103]. Its effectiveness with hydrophilic molecules like riboflavin makes it particularly suitable for TE-CXL, improving the delivery of riboflavin into the corneal stroma and enhancing the overall treatment efficiency [104]. A study comparing vitamin E-based isotonic riboflavin solution to an HPMC solution demonstrated that the maximum bulge of the posterior corneal surface (KVb) was significantly reduced following CXL with VE-TPGS [105]. Additionally, a TE-CXL protocol using high-flux UVA irradiation and riboflavin-vitamin E solution showed a significant reduction in Kmax at 24 months, with an average change of − 1.01 ± 1.22 D [106]. Ribostat CXLO, a formulation combining riboflavin and sodium iodide (NaI), has demonstrated uniform stromal riboflavin saturation sufficient for effective CXL in preclinical studies. Animal experiments have shown that Ribostat CXLO achieves consistent penetration into the rabbit corneal stroma, reaching concentrations adequate for effective CXL [107]. Additionally, the inclusion of NaI in the Ribostat CXLO solution significantly enhances riboflavin concentration within the corneal stroma, further optimizing its potential for successful CXL outcomes [108]. Clinically, in a cohort of 592 eyes, Ribostat CXLO treatment resulted in sustained improvements in vision, higher-order aberrations, and corneal topography over a two-year period without any decline in efficacy [109]. Building on these advances, a new riboflavin formulation has been shown to effectively enhance transepithelial permeability in human corneal epithelium. This formulation, composed of 1,2-dioleoyl-3-dimethylammonium-propane (DODAP) and isostearic acid (ISA) (DODAP/ISA), demonstrated permeability enhancement comparable to MedioCross TE [110]. Additionally, the TE electrical resistance (TEER) value of the DODAP/ISA formulation was nearly twice that of MedioCross TE, indicating lower cytotoxicity. Furthermore, a formulation containing 2.5% riboflavin and 0.25% DODAP/ISA significantly increased corneal epithelial permeability, while maintaining a similar TEER value. It holds potential for developing a safer and more efficient riboflavin eye drop formulation. These innovations are a key step in addressing TE-CXL limitations by improving riboflavin delivery and treatment results.

A recent addition to advanced riboflavin formulations is RitSight, a hypotonic 0.22% riboflavin solution engineered to enhance stromal diffusion in TE-CXL [111]. RitSight is supplied in a PE-LD bottle with a calibrated dropper tip, which allows adjustment of the drop size and therefore enables more consistent control of riboflavin delivery and absorption. Real-time monitoring studies have shown that RitSight achieves the highest corneal riboflavin concentration among currently available epi-on formulations, with riboflavin delivery approximately 4.5 times higher than that of other ophthalmic solutions tested [102]. RitSight achieved substantial topographic improvement, with all treated patients demonstrating Kmax flattening of at least 3.8 D during a 2-year follow-up [112]. Increasing the concentration of riboflavin in the soaking solution represents an effective strategy for improving stromal penetration through the intact epithelium and for reducing impregnation time, and RitSight applies this principle while maintaining low viscosity to facilitate diffusion. Together, these features position RitSight as one of the most promising next-generation riboflavin formulations for optimizing TE-CXL performance.

### Norepinephrine (NE)

NE has recently been studied as a new enhancer for TE-CXL. NE is a hormone synthesized and secreted by postganglionic sympathetic neurons and noradrenergic neurons in the brain [124]. It has been reported to weaken epithelial barriers by reducing tight junction expression [125]. Based on this, Liu et al. hypothesized that applying low doses of NE to the ocular surface could help riboflavin overcome the epithelial barrier and penetrate the stroma. Their mouse experiments yielded promising results, showing that NE-treated corneas achieved CXL effects comparable to those with Peschke TE [126]. However, due to the hydrophilic nature of NE, it struggles to pass through the hydrophobic corneal epithelium. To address this, they used subconjunctival injection to deliver NE, which resulted in a longer application time as NE and riboflavin could not be administered simultaneously. Although these findings are promising, further studies are needed to confirm the safety, efficacy, and practicality of NE in TE-CXL. If validated, NE could become an effective alternative enhancer for facilitating riboflavin penetration and improving CXL outcomes.

### Topical anesthetics

Topical anesthetics have also been introduced to improve patient comfort during TE-CXL procedures and to enhance riboflavin penetration. Proparacaine, commonly used at a concentration of 0.5%, is widely employed in ophthalmology due to its low irritation. It is applied before riboflavin administration to thin the tear layer to disrupt the mucin layer, and weaken the epithelium to facilitate riboflavin absorption [96]. In addition to proparacaine, tetracaine is also used, albeit at varying concentrations. A 3-year retrospective study reported that using 1% tetracaine combined with 0.1% riboflavin in TE-CXL resulted in a Kmax increase of 1.1 D, with 55% of patients showing KC progression [121]. Similarly, rabbit studies found that TE-CXL using 0.5% tetracaine resulted in less corneal stiffening compared to other common CXL methods [83]. In response to these findings, Chen et al. reduced the tetracaine concentration to 0.1% and used 0.5% riboflavin, yielding more favorable results [119]. When combined with other permeability enhancers, such as BAC, topical anesthetics can further enhance riboflavin penetration [76]. This synergistic effect allows riboflavin to more effectively reach the corneal stroma, improving the efficiency of TE-CXL. Nevertheless, despite short-term gains in penetration and comfort, biomechanical stiffening remains weaker than with S-CXL protocols, and the use of topical anesthetics in TE-CXL is not yet widely adopted in clinical practice.

### Iontophoresis-assisted delivery

Iontophoresis is a noninvasive technique that uses continuous weak currents to convey the medical molecules into tissue [127], with a long history of application across various medical fields, including gynecology and dermatology [128–130]. Iontophoresis has been widely recognized for its therapeutic benefits. In ophthalmology, this technique has been explored as a drug delivery technique since 1908 [131]. It has since been recognized as an effective method for transcorneal drug delivery to corneal tissues and the aqueous humor, and it may have potential for treating anterior segment diseases such as keratitis, glaucoma, dry eye syndrome, corneal ulcers, and ocular inflammation [127, 132]. In recent years, iontophoresis has also been investigated for its potential application in CXL, where it facilitates the penetration of riboflavin into the corneal stroma. Due to its small molecular size and ionic nature, riboflavin is well-suited for iontophoresis-assisted delivery, allowing for deeper stromal penetration while preserving epithelial integrity. This technique, known as iontophoresis-assisted transepithelial CXL (I-CXL), significantly shortens treatment time and avoids epithelial removal compared to S-CXL [133, 134]. The procedure typically employs a specialized constant current generator (I-ON XL) set to 1 mA, delivering a total dose of 5 mA over 5 min. This is followed by UVA irradiation at a wavelength of 370 nm for 9 min at an intensity of 10 mW/cm^2^, maintained at a distance of 45 mm [135]. The riboflavin solution used for I-CXL contains 0.1% riboflavin and is free of dextran and sodium chloride. To enhance its effectiveness, Tris and EDTA are added as enhancers.

A study by Mastropasqua et al. demonstrated that I-CXL achieves riboflavin saturation in the corneal stroma at levels twice as high as conventional TE-CXL methods [136]. Additionally, UDVA showed a faster recovery in the I-CXL group compared to S-CXL at the 3-month follow-up (Table 2) [137]. Furthermore, a two-year retrospective study on high-visual-acuity patients under the age of 25 suggested that I-CXL is a beneficial treatment option for young patients with KC [138]. Moreover, although I-CXL demonstrated superiority in reducing corneal thinning and improving visual quality compared to S-CXL and iontophoresis with epithelial removal (I-SCXL), these advantages must be balanced against concerns regarding its long-term durability, as progression rates remain higher than those reported for S-CXL [139]. This concern is highlighted by a prospective study on 32 patients with stage I–III KC classified according to the ABCD progression system, which found that while I-CXL effectively halted corneal ectasia progression and maintained stable UDVA and CDVA over 5 years, 7 patients (25.9%) still experienced progression [140]. In contrast, a 5-year follow-up study in pediatric patients reported no progression in the S-CXL group [141]. Furthermore, the longest available 7-year study found a progression rate of 26% in I-CXL, albeit with a limited cohort of 19 patients, whereas S-CXL demonstrated a significantly lower progression rate of 7.4% under the same parameters [142, 143]. Wu et al. reported 5-year outcomes of two continuous cycles of iontophoresis (EI-CXL), a modified technique, demonstrating its effectiveness in stabilizing progressive KC. The study also reported a lower postoperative complication rate in EI-CXL (8.08%) compared to S-CXL (11.7%) [144]. These findings consistently highlight that although I-CXL may provide short-term visual and comfort benefits, S-CXL remains superior in terms of long-term efficacy and durability. Larger long-term studies are needed to further validate these findings.

**Table 2:** Comparative studies of I-CXL and S-CXL: key clinical outcomes

Author	Year	Eyes (I/S)	Follow-up	UVA irradiation and duration	Mean ∆Kmax (D)		Mean ∆CDVA (logMar)		Mean DL (µm)		Key results
					I-CXL	S-CXL	I-CXL	S-CXL	I-CXL	S-CXL	
Bilgihan K, et al. [151]	2022	18/18	4 yrs	9 mW/cm^2^ for 13.3 min	− 0.57	− 1.53	− 0.02	− 0.24	264.50 (1 mo)	287.12 (1 mo)	DAI-CXL and S-CXL demonstrate comparable 4-year outcomes
Cantemir A, et al. [137]	2017	40/40	36 mos	10 mW/cm^2^ for 9 min	− 0.90	− 1.20	0.08	0.10	134 (1 mo)	286 (1mo)	I-CXL had 2.5% progression; S-CXL 20% adverse-events incidence
Vinciguerra P, et al. [139]	2019	20/20	2 yrs	10 mW/cm^2^ for 9 min	− 0.40	0	− 0.10	− 0.08	NR	NR	I-CXL reduces corneal thinning and aberrations
Rossi S, et al. [145]	2018	10/10	12 mos	10 mW/cm^2^ for 10 min	− 0.87^*^	− 1.50^*^	− 0.13	− 0.13	NR	NR	UDVA and CDVA improved significantly in all groups
Lombardo M, et al. [148]	2017	22/12	12 mos	10 mW/cm^2^ for 9 min	− 0.52	− 0.82	− 0.10	− 0.03	NR	NR	1 eye (8%) had sterile corneal infiltrates in S-CXL
Alqudah N, et al. [158]	2025	54/53	12 mos	3 mW/cm^2^ for 30 min	0.11	− 0.59	0.04	0.05	NR	NR	5.5% of patients had severe haze in S-CXL
Lombardo M, et al. [159]	2019	22/12	24 mos	10 mW/cm^2^ for 9 min	− 1.05	− 1.51	− 0.08	− 0.02	NR	NR	S-CXL achieved greater corneal apex flattening than I-CXL
Buzzonetti L, et al. [160]	2019	20/20	3 yrs	10 mW/cm^2^ for 9 min	2.90	0.80	0.00	− 0.1	NR	NR	S-CXL halted 75% progression; I-CXL slowed 50% of cases
Atia R, et al. [161]	2018	30/30	6 mos	10 mW/cm^2^ for 9 min	0.20	0.10	− 0.07	− 0.01	NR	NR	S-CXL induces thinner, regular epithelium
Jia HZ, et al. [162]	2017	17/13	12 mos	3 mW/cm^2^ for 30 min	− 1.30	− 2.21	− 0.13	− 0.11	NR	NR	I-CXL is unable to completely replace S-CXL
Jouve L, et al. [163]	2017	40/40	24 mos	10 mW/cm^2^ for 9 min	0.20	− 1.10	− 0.07	− 0.09	216 (1 mo)	291 (1 mo)	I-CXL had 20% failure rate; S-CXL had 7.5%
Bikbova G, et al. [164]	2016	76/73	24 mos	3 mW/cm^2^ for 30 min	− 0.97	− 2.15	0.26	0.30	172 (14 d)	292 (14 d)	Four patients retained permanent hyperreflective stromal haze in S-CXL
Vinciguerra P, et al. [165]	2016	20/20	1 yrs	10 mW/cm^2^ for 9 min	− 0.31	− 1.05	− 0.10	− 0.05	Anterior stroma reflectance increase	Standard depth	I-CXL may match S-CXL in stabilizing KC progression
Bouheraoua N, et al. [166]	2014	15/15	6 mos	10 mW/cm^2^ for 9 min	0.40	− 1.80	− 0.06	− 0.05	212 (1 mo)	302.8 (1 mo)	I-CXL caused less damage to corneal nerves and keratocytes

^*^The values are estimated from the figure and may not represent exact measurements; ∆ represents the difference from the baseline; I, I-CXL = transepithelial corneal cross-linking with iontophoresis; DAI-CXL = transepithelial diluted alcohol and iontophoresis-assisted corneal cross-linking; S, S-CXL = standard corneal cross-linking with Dresden protocol; D = diopter; Kmax = maximum keratometry; UDVA = uncorrected distance visual acuities; CDVA = corrected distance visual acuities; DL = demarcation line; NR = none report; KC = keratoconus; mo = month; yrs = years; d = day

Some studies have suggested that I-CXL and S-CXL offer comparable therapeutic effects and refractive stability, with findings indicating that their CXL efficacy is nearly equivalent [145–147]. In contrast, most clinical studies comparing I-CXL and S-CXL indicate that I-CXL has a slightly less pronounced effect on Kmax and a shallower demarcation line compared to S-CXL (Table 2). At 12 months, Kmax reduction was − 0.52 ± 1.30 D for I-CXL, slightly less than − 0.82 ± 1.20 D with the S-CXL [148]. Moreover, 27% of patients experienced further disease progression after I-CXL and required retreatment with S-CXL, which has been proven effective in halting KC progression following I-CXL failure [149, 150]. To enhance its efficacy, several modifications to the traditional I-CXL protocol have been proposed. Among these, the combination of I-CXL with dilute alcohol (10% ethanol), previously discussed in the alcohol-assisted CXL section, has shown promising results [151, 152]. Additionally, enhanced fluence and pulsed UV-A light protocols, with optimized energy at 7 J/cm^2^ (18 mW/cm^2^, pulsed for 6.28 min over a total irradiation time of 12.56 min), as well as extending the iontophoresis duration from 5 to 10 min, both of which demonstrated improved CXL efficiency [153–155]. Furthermore, DI-CXL, which utilizes a double-cycle iontophoresis technique to enhance riboflavin penetration, has demonstrated improved corneal stabilization, with long-term outcomes comparable to S-CXL [156]. Nevertheless, these refinements remain under investigation, and questions persist regarding the long-term reliability of I-CXL. While technical innovations have brought the efficacy of I-CXL closer to that of S-CXL, evidence still suggests that its stability is less consistent over extended follow-up. Potential complications, such as epithelial damage during suction ring placement and the risk of iontophoresis suction loss leading to riboflavin wastage and increased procedural costs, also remain concerns [157]. Therefore, although I-CXL represents an encouraging avenue for reducing patient discomfort and expanding treatment options, S-CXL continues to serve as the more reliable benchmark for halting KC progression.

### UVA enhancement strategies

Accelerated CXL (A-CXL) is based on the Bunsen-Roscoe law of reciprocity, which states that the same total energy can be achieved by increasing UVA intensity while reducing exposure time [167]. The combination of a transepithelial approach with an accelerated protocol has led to the development of transepithelial accelerated CXL (TE-ACXL) in recent years [168]. Compared to traditional TE-CXL, TE-ACXL shortens treatment time while maintaining the total radiation dose, potentially improving patient comfort and reducing procedural risks. Additionally, TE-ACXL protocols often incorporate enhanced riboflavin formulations or iontophoresis to improve riboflavin penetration, addressing a key limitation of conventional TE-CXL. Currently, commonly used TE-ACXL protocols include UVA fluence of 6 mW/cm^2^ for 15 min [169, 170], 9 mW/cm^2^ for 10 min [171–173], 18 mW/cm^2^ for 5 min [174], and 30 mW/cm^2^ for 3 min [175, 176], all delivering a total energy dose of 5.4 J/cm and proven to be safe and effective (Table 3). Madeira et al. have reported that TE-ACXL achieves similar outcomes to S-CXL in terms of corneal stabilization and refractive parameters and compared the traditional 3 mW/cm^2^ for 30 min with 6 mW/cm^2^ for 15 min found similar efficacy in corneal stabilization, with the latter offering a shorter treatment time and improved patient comfort without compromising safety [177]. However, these encouraging short-term findings must be interpreted with caution given the accumulating long-term evidence. A two year outcomes study reported that 11% to 33% of patients had disease progression with no complications during TE-ACXL [173]. Long-term follow-up studies also suggest that TE-ACXL may have a higher risk of KC progression compared to S-CXL. For instance, 67.9% of patients experienced further KC progression within four years after undergoing TE-ACXL (6 mW/cm^2^ for 15 min) [170]. Similarly, Henriquez et al. conducted a five-year follow-up study comparing the effects of TE-ACXL using 18 mW/cm^2^ for 5 min and S-CXL. They found that the progression rate was 9.37% in the TE-ACXL group, whereas no progression was observed in the S-CXL group (Table 4) [141]. These data indicate that, despite offering greater patient comfort and shorter procedure times, TE-ACXL still provides less durable disease control than S-CXL.

**Table 3:** TE-ACXL protocol summary (Sorted by total fluence)

Authors	UVA Power (mW/cm^2^)	Irradiation time (min)	Total fluence (J/ cm^2^)
Vilares-Morgado R, et al. [170]	6	15	5.4
Mazzotta C, et al. [183]	9	10	5.4
Hashemi H, et al. [184]	18	5	5.4
Ishii H, et al. [175]	30	3	5.4
Mazzotta C, et al. [154]	18	6.28	7
Kır MB, et al. [178]	45	2.40	7
Zhang X, et al. [185]	45	5.20 (1 s on/1 s off)	7.2

**Table 4:** Comparative studies of TE-ACXL and S-CXL: key clinical outcomes

Author	Year	Eyes (TE-A/S)	Follow-up	UVA power (mW/cm^2^)	Irradiation time (min)	Total Fluence (J/ cm^2^)	Mean ∆Kmax (D)		Mean ∆CDVA (logMar)		Key foundings
							TE-ACXL	S-CXL	TE-ACXL	S-CXL	
Henriquez MA, et al. [141]	2020	32/46	5 yrs	18	5	5.4	− 0.28	− 1.63	0.06	0.09	TE-ACXL showed 9.37% KC progression rate
Madeira C, et al. [177]	2019	16/10	12 mos	6	15	5.4	− 2.13	0.78	− 0.04	0.02	TE-ACXL and C-CXL were similarly effective
Henriquez MA, et al. [186]	2017	36/25	12 mos	18	5	5.4	0.10	− 0.94	0.09	0.06	KC progression: 5.6% in TE-ACXL, 12% in S-CXL

∆ represents the difference from the baseline; TE-A, TE-ACXL = transepithelial accelerated corneal cross-linking; S, S-CXL = standard corneal cross-linking with Dresden protocol; D = diopter; Kmax = maximum keratometry; CDVA = corrected distance visual acuities; KC = keratoconus; mos = months; yrs = years

Some studies have also explored enhanced energy protocols, such as 7 J/cm^2^ (18 mW/cm^2^ for 6.28 min) [154], and 45 mW/cm^2^ for 2.40 min to further improve CXL efficacy while minimizing corneal damage. Although high-intensity TE-CXL (45 mW/cm^2^ for 2.40 min) does not significantly improve CDVA, SE, or topographic parameters, it has been shown to effectively halt KC progression over two years [178]. High-fluence TE-ACXL protocols delivering 7.2 J/cm^2^ have shown encouraging results in improving visual acuity and limiting disease progression. For instance, a pulsed illumination regimen using 45 mW/cm^2^ for 5 min and 20 s (1 s on/1 s off) significantly improved CDVA, with only 3.5% of patients showing KC progression, compared to 35% in the standard accelerated group [179]. A similar protocol demonstrated improvements in both UCVA and CDVA at 6-month follow-up [180]. However, TE-ACXL still faces challenges in achieving the same depth and biomechanical strengthening as S-CXL. TE-ACXL generally achieves a shallower CXL depth compared to S-CXL due to limited riboflavin penetration [181]. This increases the risk of KC progression, particularly in patients with thinner corneas. While TE-ACXL has shown short-term corneal stabilization, its long-term effects on corneal biomechanics remain unclear, particularly in younger patients who may require stronger cross-linking.

TE-ACXL has emerged as a promising alternative to traditional TE-CXL by maintaining the total radiation dose while shortening treatment time. However, its clinical adoption remains limited, primarily due to concerns regarding safety, uncertain long-term outcomes, and the lack of standardized treatment protocols. A case report indicated that 64% of patients developed complications within the first postoperative week after TE-ACXL, raising concerns about its safety and consistency, which contradicts the purpose of preserving the epithelium and further reduces cross-linking efficacy [182]. Additionally, there are currently only a limited number of clinical studies comparing TE-ACXL and S-CXL, resulting in a lack of well-defined treatment parameters for TE-ACXL. Further investigations are required to refine its procedural guidelines, establish consensus on optimal treatment parameters, and evaluate its long-term clinical efficacy before TE-ACXL can be considered a reliable alternative to S-CXL.

### Oxygen enhancement strategies

Oxygen plays a key role in the CXL process, as it is directly involved in the formation of reactive oxygen species (ROS) during UV-A irradiation in the presence of riboflavin [187]. These ROS mediate the CXL of stromal collagen, leading to biomechanical stiffening [188]. However, oxygen mainly supports the regeneration of riboflavin during UV-A exposure, so its contribution to enhancing cross-linking is limited by its low solubility and rapid consumption within the stroma [189]. Within a safe physiological range, increasing environmental oxygen availability may modestly enhance cross-linking efficiency, but excessive oxygen exposure carries a theoretical risk of promoting radical quenching or even cellular damage [190]. Despite these biochemical constraints, several studies have explored whether elevating oxygen availability can augment the CXL effect. An ex vivo study demonstrated that increasing the oxygen concentration from 20.9% (ambient air) to 90% (high-oxygen environment) led to a 4.3-fold increase in oxygen flux, significantly enhancing the CXL effect and highlighting the critical role of oxygen in the CXL process [191]. Similarly, another study found that increasing oxygen availability during TE-CXL slowed the KC progression significantly in one year [192]. The corneal demarcation line induced by high-oxygen TE-CXL had an average depth of 414 ± 80.91 μm, reaching 89% of the total corneal thickness and indicating that high-oxygen environments may enhance stromal penetration [193]. Additionally, a recent systematic review found that increasing oxygen availability during epithelium-on CXL was associated with modest improvements in visual acuity and corneal flattening without major adverse events [194]. Overall, these findings suggest that oxygen may help improve some aspects of TE-CXL, but the actual benefit is limited by stromal oxygen availability and still needs to be confirmed in larger, long-term studies.

Currently, most clinical studies on oxygen enhancement in TE-CXL combine it with TE-ACXL to improve CXL efficacy. A one-year follow-up clinical study indicated that TE-ACXL with oxygen supplementation can effectively halt the progression of corneal ectasia [195]. To further improve oxygen availability, most studies use oxygen goggles as a supplemental oxygen delivery system. These devices help sustain high stromal oxygen levels by creating a localized high-oxygen environment, thereby enhancing the effectiveness of the CXL process. Cronin et al. utilized oxygen goggles combined with pulsed UV-A irradiation (1 s on, 1 s off) and found that this approach improved CDVA, while Kmax remained largely unchanged [196]. However, other studies also used oxygen goggles and reported that this method resulted in improvements in both Kmax and CDVA [48, 197]. Additionally, the study by Al Saidi showed that TE-CXL with supplemental oxygen resulted in modest improvements in CDVA after one year [198]. In summary, a high-oxygen environment has been shown to effectively enhance TE-ACXL outcomes, with studies generally reporting positive results. The combination of oxygen supplementation with TE-ACXL appears to be a promising future direction, potentially offering greater efficacy than TE-CXL alone. However, current clinical studies have relatively short follow-up periods, and further research is needed to confirm long-term benefits.

### Microneedle-based systems

Microneedle (MN) is a novel drug delivery system that consists of a micron-scale needle-like structure used for transdermal or trans-tissue delivery of drugs, genes, or other therapeutic substances [199]. In ophthalmology, MN offer a minimally invasive approach (length < 1 mm), enabling targeted drug delivery with reduced tissue trauma and enhanced localization [200]. This makes them a promising treatment option for posterior segment diseases such as age-related macular degeneration, uveitis, retinal vascular occlusion, and diabetic retinopathy [200]. In the past two years, the use of microneedles to enhance riboflavin penetration into the cornea has emerged as an innovative technique in corneal cross-linking, addressing the issue of insufficient riboflavin concentration in TE-CXL. For instance, Hu et al. have developed a novel responsive porous MN that enables the precise delivery of riboflavin to the target area to enhance corneal biomechanical properties without removing the epithelium [201]. The MN system consists of three distinct layered structural components: poly (N-isopropylacrylamide) (PNIPAM), graphene oxide (GO), and gelatin loaded with riboflavin, allowing riboflavin to be continuously delivered into the corneal stroma through microchannels. Similarly, Yang et al. conducted an ex vivo rabbit study using a customized CXL approach with MN. The protocol involved MN, supplementation with 5 mg/mL riboflavin, and exposure to 9 mW/cm^2^ UVA for 10 min, achieving an efficacy comparable to S-CXL [202]. Since the penetration depth of the MN was approximately 25 μm, they were able to pierce the corneal epithelium without damaging the stromal layer, allowing for rapid postoperative recovery of the corneal tissue. Currently, MN-assisted CXL remains in the research stage, but it presents a promising novel therapeutic approach. This technique holds potential for customized treatment of corneal diseases, offering a minimally invasive and efficient alternative to conventional CXL methods.

### Nanotechnology delivery systems

Researchers have explored nanocarrier-based delivery systems, including liposomes, cyclodextrins, and nanoemulsions, to enhance riboflavin absorption and penetration. Liposomal riboflavin formulations encapsulate riboflavin within nanoscale lipid bilayers, enabling prolonged retention on the ocular surface and controlled release into the corneal stroma. Ahmad et al. reported that liposomal formulations improve riboflavin stability, protecting it from photodegradation and enhancing its bioavailability [203]. However, Kandzija et al. reported that encapsulating riboflavin in liposomes did not significantly enhance riboflavin penetration into the bovine cornea [204]. Similarly, cyclodextrin-based carriers increase riboflavin solubility and permeability, facilitating deeper diffusion without disrupting the epithelial layer. Morrison et al. demonstrated that in vitro diffusion experiments using bovine corneas showed β-cyclodextrin increased riboflavin solubility to 0.12–0.19 mg/mL [205]. Additionally, when cyclodextrin, chitosan, and arginine are incorporated into the formulation, the solubility of riboflavin is significantly enhanced [206]. Furthermore, nanoemulsions have been developed to improve riboflavin delivery by enhancing corneal penetration and prolonging retention. Sodium riboflavin-5-phosphate exhibits stronger adhesion to positively charged nanostructured lipid carriers (NLCs) and the negatively charged corneal surface, thereby improving transcorneal penetration [207]. Bottos et al. demonstrated that riboflavin-5-phosphate nanoemulsion can penetrate the corneal epithelium and diffuse into the stroma [208]. These nanocarrier-based delivery systems offer promising strategies to enhance riboflavin penetration and efficacy in TE- CXL. However, most of the current evidence comes from laboratory or animal studies, and clinical data are still limited. More research is needed to confirm their safety and long-term effectiveness before they can be widely used in practice.

### Ultrasound-assisted treatment

Unlike traditional TE-CXL methods that rely on chemical permeability enhancers or iontophoresis, ultrasound propagates through liquids by displacing fluid molecules, generating a stable, unidirectional flow that facilitates substance penetration and distribution within tissues. Additionally, ultrasound induces cavitation, where microbubbles form and collapse, producing mechanical forces that alter the surrounding tissue structure, thereby enhancing therapeutic effects [209, 210]. As a result, ultrasound utilizes high-frequency sound waves to temporarily disrupt epithelial tight junctions, increasing corneal permeability. In an animal study conducted by Lamy et al. ultrasound-assisted treatment increased the local diffusion of riboflavin into the corneal stroma [211]. However, when compared to epithelium-off eyes, which did not receive ultrasound treatment, the non-ultrasound-treated epithelium-off group exhibited greater riboflavin penetration into the stroma. Ultrasound-assisted treatment presents potential advantages, as it may help avoid the cytotoxic effects associated with certain chemical permeability enhancers. However, current evidence is limited, and further studies are needed to determine its clinical feasibility and effectiveness.

### Theranostic-guided TE-CXL

Theranostic-guided TE-CXL represents a new personalized approach that integrates real-time diagnostic monitoring of stromal riboflavin concentration with adaptive UV-A irradiation [212, 213]. By calculating a riboflavin score and a theranostic score, this technology aims to overcome the traditional limitations of TE-CXL, particularly insufficient riboflavin penetration and UV light mediated photoactivation of the substance [214]. A Randomized multicenter trial for Guided CXL Optimization (ARGO) showed a positive theranostic response achieved an average Kmax flattening of 1.3 ± 0.9 D, which is comparable to the outcomes typically reported for S-CXL (around –1.3 ± 1.4 D) and notably higher than the effect observed with most currently available TE-CXL techniques (approximately –0.3 ± 1.5 D). These findings highlight that achieving adequate stromal riboflavin levels and effective UV photoactivation remain the main determinants of CXL efficacy. Building on this, theranostic guidance provides real-time feedback on these parameters, helping to individualize TE-CXL treatment and potentially optimize outcomes for patients with keratoconus.

## Discussion

TE-CXL offers significant advantages by preserving the epithelium and reducing postoperative discomfort. However, several challenges limit its widespread clinical application. One of the primary limitations is poor riboflavin penetration, as the intact corneal epithelium acts as a barrier, reducing the cross-linking depth and biomechanical strengthening. Various methods, such as chemical enhancers, iontophoresis, and UVA enhancement, have been explored to improve penetration, yet none have become standard due to inconsistent outcomes. Beyond these biochemical limitations, variations in UVA fluence and irradiation patterns may also influence TE-CXL performance. Higher fluence settings may partly compensate for reduced oxygen availability, while pulsed-light protocols allow intermittent oxygen replenishment during the “off” cycles, potentially enhancing the oxidative effect. However, increasing fluence and using pulsed modes may also influence a deeper penetration of oxidative damage, which raises safety concerns in thinner corneas. Although early data suggest these adjustments can improve CXL efficiency in TE-CXL treatments, their clinical benefits remain inconsistent and do not yet match those of S-CXL.

Another major challenge is the variability in clinical results. Unlike S-CXL, which benefits from well-established protocols, TE-CXL lacks standardized treatment guidelines. Differences in riboflavin formulations, UVA exposure settings, and individual corneal properties contribute to the inconsistent long-term stability. Some studies report higher rates of KC progression following TE-CXL, and epithelial irregularities or delayed healing have been documented despite the procedure’s less invasive nature.

In summary, TE-CXL represents a promising and less invasive alternative, but current evidence does not support it as equivalent to S-CXL. While it reduces postoperative pain and recovery time, this benefit comes with a trade-off in terms of effectiveness and long-term stability. Compared with S-CXL, TE-CXL may be less consistent in halting KC progression and achieving lasting visual improvements. Future efforts should focus on optimizing riboflavin formulations for improved penetration, refining iontophoresis and UVA parameters, and conducting large-scale, long-term clinical trials. Emerging technologies such as theranostic-guided TE-CXL may help address some of these limitations by providing real-time feedback on stromal riboflavin penetration and treatment efficacy. However, further clinical validation is still required before these approaches can be fully integrated into routine practice. Only with standardized protocols and stronger clinical validation can TE-CXL evolve into a more reliable option for KC management.

## Data Availability

No new data were generated or analyzed in this study. Data sharing is not applicable to this article as it is a review based on previously published studies.
